# Association of peripheral CD4⁺/CD8⁺ T-cell ratio with SYNTAX score and coronary stenosis severity in patients with coronary artery disease

**DOI:** 10.1016/j.clinsp.2026.101211

**Published:** 2026-10-15

**Authors:** Wenhao Li, Chunzi Jin, Meina Piao, Xiang Li, Guangming Jin

**Affiliations:** Cardiology Department, Affiliated Hospital of Yanbian University (Yanbian Hospital), Yanji, Jilin, China

**Keywords:** T-lymphocytes, SYNTAX score, Coronary stenosis, Coronary artery disease, CD4/CD8 ratio

## Abstract

**Background:**

Coronary Artery Disease (CAD) is characterized by chronic immune-inflammatory activation. The relationship between the peripheral CD4+/CD8+ T-cell ratio and coronary stenosis severity remains unclear. We investigated its association with SYNTAX score and potential as a complementary marker.

**Methods:**

CD4+ and CD8+ T-cell counts were measured by flow cytometry in 297 patients with CAD and 181 controls. Coronary stenosis was assessed by angiography and SYNTAX score. Multivariable linear regression was used for SYNTAX score, and logistic regression for moderate-to-severe stenosis (SYNTAX > 23). Models were adjusted for age, sex, BMI, hypertension, diabetes, smoking, HbA1c, hs-CRP, creatinine, and medications. Benjamini-Hochberg FDR was applied. ROC analysis assessed incremental value beyond risk factors.

**Results:**

CD4+ counts and the CD4+/CD8+ ratio were higher, and CD8+ counts lower, in CAD patients, especially in acute coronary syndromes. In unadjusted analyses, SYNTAX score correlated positively with CD4+ count and the CD4+/CD8+ ratio and negatively with CD8+ count (weak to moderate). After adjustment, the CD4+/CD8+ ratio remained independently associated with SYNTAX score (β = 4.88, 95% CI 1.42–8.30, p = 0.006). For SYNTAX > 23, the ratio showed limited discrimination (AUC = 0.598, 95% CI 0.526–0.670; sensitivity 0.587, specificity 0.552 at cutoff 1.90). Adding the ratio to conventional risk factors showed no substantial incremental value (ΔAUC = 0.010, DeLong p = 0.43, NRI = 0.082, IDI = 0.006).

**Conclusions:**

Peripheral T-cell imbalance is associated with greater angiographic coronary complexity in CAD. However, the CD4+/CD8+ ratio has limited discriminatory ability and should be considered a complementary candidate marker, not a standalone diagnostic or predictive tool. Larger prospective multicenter studies are required.

## Highlights

• CD4⁺/CD8⁺ ratio is elevated in CAD patients, especially in ACS.
• CD4⁺/CD8⁺ ratio correlates positively with SYNTAX score (*r* = 0.303).
• The ratio shows moderate diagnostic value for severe stenosis (AUC = 0.833).
• It may serve as a supplementary biomarker for CAD severity assessment.

## Introduction

Coronary Artery Disease (CAD) and its complications remain the predominant causes of global morbidity and mortality.^1^ Atherosclerosis, the principal pathological substrate of CAD, is now firmly established as a chronic inflammatory disorder of the vascular wall, initiated by lipid retention and perpetuated by intricate crosstalk between innate and adaptive immune compartments.^2^ Over the past decade, accumulating evidence has substantiated that immune dysregulation not only underpins the onset of atherosclerotic lesions but also critically drives their progression toward unstable, rupture-prone phenotypes.

T-lymphocytes have emerged as pivotal orchestrators of adaptive immunity within the atherosclerotic milieu.^3^^,^^4^ Both CD4⁺ helper T-cells and CD8⁺ cytotoxic T-cells are abundantly present in human atheromata, where they modulate lesional inflammation through the release of diverse cytokines and direct cellular interactions. Among CD4⁺ subsets, Th1-polarized cells exert pro-atherogenic effects primarily via Interferon-Gamma (IFN-γ) secretion, which activates macrophages and amplifies local inflammatory cascades.^5^^,^^6^ Conversely, regulatory T-cells (Tregs) counterbalance this response by furnishing immunosuppressive cytokines, thereby conferring atheroprotection. CD8⁺ T-lymphocytes, on the other hand, contribute to plaque vulnerability through cytotoxic granule release and facilitation of necrotic core expansion.^4^^,^^7^ Collectively, the homeostatic equilibrium between pro-inflammatory and regulatory T-cell subsets is increasingly recognized as a critical determinant of atherosclerotic disease trajectory.

In healthy individuals, CD4⁺ and CD8⁺ T-lymphocytes maintain a tightly regulated numerical and functional balance. However, this equilibrium is profoundly perturbed in patients with CAD.^3^^,^^8^ Prior investigations have documented significant shifts in peripheral T-lymphocyte subpopulations among CAD patients, typified by elevated CD4⁺ counts, diminished CD8⁺ counts, and a consequent elevation in the CD4⁺/CD8⁺ ratio.^8^ While these alterations are thought to mirror systemic immune activation and progressive immune senescence accompanying coronary atherosclerosis, the precise relationship between peripheral T-cell subset imbalances and the anatomical burden of coronary stenosis ‒ particularly when quantified by contemporary angiographic scoring systems ‒ remains poorly defined.

The SYNTAX score is a well-validated angiographic instrument that comprehensively grades the anatomical complexity and severity of CAD based on the entire coronary tree. It has proven prognostic utility in predicting long-term adverse events across diverse CAD populations, including those presenting with acute coronary syndromes or undergoing complex revascularization procedures.^9^ Nevertheless, while the SYNTAX score offers robust anatomic stratification, it inherently lacks insight into the underlying immuno-inflammatory milieu ‒ a key determinant of plaque behavior and clinical outcomes. Consequently, the identification of accessible circulating biomarkers that accurately reflect the systemic immune status and correlate with anatomical disease burden holds substantial promise for refining risk stratification and guiding individualized therapeutic strategies.

Recent technological advances, including mass cytometry and single-cell transcriptomics, have unveiled distinct peripheral immune signatures associated with varying clinical presentations of CAD, further emphasizing the clinical relevance of T-cell profiling.^6^^,^^10^ Despite these insights, the majority of existing studies have examined immunological parameters and anatomical assessments in parallel rather than in an integrated fashion. Critically, few investigations have systematically interrogated the direct correlation between peripheral T-lymphocyte subset alterations and the extent of coronary stenosis using a validated, quantitative angiographic scoring system.

To address this gap, the present study was designed to evaluate the association between peripheral CD4⁺ and CD8⁺ T-cell counts, the CD4⁺/CD8⁺ ratio, and the severity of coronary stenosis as quantified by the SYNTAX score in a well-characterized CAD cohort. Furthermore, we appraised the discriminatory performance of the CD4⁺/CD8⁺ ratio for detecting moderate-to-severe coronary stenosis, aiming to ascertain its potential utility as a complementary candidate marker for CAD severity stratification. By integrating immunological phenotyping with angiographic assessment, this study endeavors to foster a more holistic understanding of CAD pathophysiology and to explore whether routine T-cell subset measurements can provide additive clinical value beyond traditional risk factors and conventional scoring systems.

## Patients and methods

### Ethics approval and consent to participate

The study was performed in accordance with the ethical standards of the 1964 Declaration of Helsinki and its later amendments or comparable ethical standards. Approval was obtained from the Ethics Committee of Affiliated Hospital of Yanbian University (Yanbian Hospital) (approval number: 2019,135, date of approval: September 15, 2019). Written informed consent was obtained from all individual participants included in the study.

### Patients

This was a prospective, cross-sectional, single-center study. Patients were consecutively enrolled from October 2019 to March 2020 at the Cardiology Department of Affiliated Hospital of Yanbian University (Yanbian Hospital). We enrolled 478 patients who were admitted to the Cardiology Department of Affiliated Hospital of Yanbian University (Yanbian Hospital) from October 2019 to March 2020. The CAD Group (CAD-G) contained 297 patients with a history of typical chest pain; signs of myocardial ischemia indicated by Electrocardiography (ECG), Doppler echocardiography; and a percentage Diameter Stenosis (%DS) ≥ 50% in at least one Coronary Artery (CA) or its major branches demonstrated by selective coronary angiography.

The control group (control-G) consisted of 181 patients with no evidence of myocardial ischemia on examination and no obvious coronary obstructive lesions confirmed by coronary angiography. The CAD-G was further divided into four groups: SAP Group (SAP-G), UAP Group (UAP-G), NSTEMI Group (NSTEMI-G), and STEMI Group (STEMI-G).

Patients with the following conditions were excluded from the study: 1) Patients with primary cardiomyopathy; 2) Patients with heart valve disease; 3) Patients who had undergone stenting or CA bypass grafting within the past 3-months; 4) Patients with combined trauma, tumors, tuberculosis, advanced liver disease, renal failure, sepsis, ongoing steroid therapy, or other inflammatory diseases.

### Quantification of CD4⁺ and CD8⁺ T-lymphocyte subsets in peripheral blood

Fasting venous blood samples (5 mL) were collected in the morning into EDTA-K2 anticoagulant tubes (Becton, Dickinson and Company, Franklin Lakes, NJ, USA). Peripheral Blood Mononuclear Cells (PBMCs) were isolated from whole blood within 2 h of collection using Ficoll-Paque PLUS density gradient centrifugation (density 1.077 g/mL, Cytiva, formerly GE Healthcare, Catalog #17–1440–03, Marlborough, MA, USA). Cell surface staining was performed using Fluorescein Isothiocyanate (FITC)-conjugated anti-human CD4 monoclonal antibody (Clone: OKT4, Catalog #317,408, BioLegend, San Diego, CA, USA) and Phycoerythrin (PE) ‒ conjugated anti-human CD8 monoclonal antibody (Clone: HIT8a, Catalog #300,908, BioLegend). Briefly, 100 μL of whole blood was incubated with optimized concentrations of the antibodies for 30-minutes at 4 °C in the dark. Following incubation, red blood cells were lysed with BD FACS™ Lysing Solution (10 × Concentrate, Catalog #349,202, Becton, Dickinson and Company) according to the manufacturer’s instructions. After washing with Phosphate-Buffered Saline (PBS), the stained cells were fixed with 1% paraformaldehyde. Data acquisition was performed on a BD FACSCalibur™ flow cytometer (Becton, Dickinson and Company) equipped with a 488 nm argon-ion laser and a 635 nm red diode laser, capable of four-color fluorescence analysis. A minimum of 10,000 events were acquired per sample. The absolute counts (cells/μL) of CD4⁺ and CD8⁺ T-cell subsets were determined using BD TruCOUNT™ Tubes (Catalog #342,445 for the CD4 FITC/CD8 PE/CD3 PerCP kit, Becton, Dickinson and Company) containing a lyophilized pellet of fluorescent beads of known concentration as an internal standard, following the manufacturer’s protocol. Data analysis was performed using CellQuest™ Pro software (Version 6.0, Becton, Dickinson and Company). Strict quality control was maintained by running BD™ CS&T beads (Cytometer Setup and Tracking beads, Becton, Dickinson and Company) daily to ensure consistent performance of the instrument’s optics, electronics, and fluids. The assay showed excellent precision, with intra-assay and inter-assay Coefficients of Variation (CVs) of < 5% and < 8%, respectively, as determined in our laboratory.

### Coronary angiography and assessment of the extent of CAD

A Siemens angiography system (SIEMENS, ARTIS pheno-164,133) with a flat-panel digital detector was used to generate coronary angiograms. Coronary angiography was performed using the Judkins technique,^11^ and a combination of visual assessment and quantitative coronary angiography system measurements was used to determine the %DS. Patients with a %DS ≥ 50% were included in the CAD-G, while patients with a %DS < 50% were included in the control-G. The SYNTAX score was used to assess the extent of coronary stenosis.^9^ The SYNTAX score was calculated for all patients in the CAD-G using the online SYNTAX Score calculator 2.28, available at www.syntaxscore.org. The assessment was performed independently by two experienced interventional cardiologists who were blinded to the patients’ clinical history, laboratory results, and T-cell subset data. Each lesion with a diameter stenosis ≥ 50% in vessels ≥ 1.5 mm was included in the score calculation. In cases of disagreement between the two assessors, a consensus was reached through discussion with a third senior interventional cardiologist. The interobserver agreement was excellent, with an Intraclass Correlation Coefficient (ICC) of 0.94 (95% CI 0.91‒0.97) based on a preliminary analysis of 20 randomly selected angiograms.

### Statistical analysis

Statistical analyses were performed using R software version 4.3.1 (R Foundation for Statistical Computing, Vienna, Austria). The pROC package was used for ROC curve analysis, and the p.adjust function was used for Benjamini-Hochberg false discovery rate correction; *q* < 0.05 was considered statistically significant. Normally distributed variables are presented as mean ± standard deviation and compared using one-way ANOVA followed by Tukey’s post hoc test. Non-normally distributed variables are presented as median (interquartile range) and compared using the Kruskal-Wallis test followed by Dunn’s post hoc test. Categorical variables were compared using the Chi-Square test or Fisher’s exact test when appropriate.

Correlation analyses were performed using Pearson or Spearman correlation according to data distribution. Correlation coefficients and 95% confidence intervals were reported. The strength of correlation was interpreted as weak (< 0.30), moderate (0.30–0.50), or strong (> 0.50).

Multivariable linear regression was used to evaluate the association between T-cell subsets and SYNTAX score. Because CD4⁺ count, CD8⁺ count, and the CD4⁺/CD8⁺ ratio are collinear, they were entered into separate models. Model 1 was unadjusted. Model 2 adjusted for age, sex, BMI, hypertension, diabetes, smoking, HbA1c, hs-CRP, and creatinine. Model 3 additionally adjusted for medication use, including statins, ACE inhibitors/ARB, beta-blockers, and antiplatelet agents. Logistic regression was used to assess the association between the CD4⁺/CD8⁺ ratio and SYNTAX score > 23, with results reported as odds ratios and 95% confidence intervals.

ROC analysis was restricted to patients with CAD and was performed using the pROC package. The AUC and 95% confidence interval were calculated using the DeLong method. The optimal cutoff was determined by the Youden index. Sensitivity, specificity, positive predictive value, and negative predictive value were reported. To assess incremental value, a base clinical model was compared with a model adding the CD4⁺/CD8⁺ ratio using DeLong’s test, net reclassification improvement, and integrated discrimination improvement. A two-sided p-value < 0.05 was considered statistically significant unless otherwise specified.

## Results

### Study population

The groups did not differ in terms of age, sex, or body mass index. The prevalence rates of hypertension and diabetes were significantly higher in the CAD-G than in the control-G (Table 1).

**Table 1:** Comparison of baseline characteristics between the control group and CAD group.

Index	Control (n = 181)	CAD (n = 297)	p-value
Age (years)	63.74 ± 8.51	62.91 ± 10.24	0.17
Female sex (%)	35.12	33.85	0.31
BMI (kg/m²)	24.52 ± 3.08	24.86 ± 3.29	0.35
Hypertension (%)	42.61	65.24	<0.01
Diabetes (%)	10.65	31.22	<0.01
Smoking (%)	22.07	44.36	<0.01

Notes: CAD, Coronary Artery Disease; BMI, Body Mass Index. Continuous variables are presented as mean ± standard deviation or median (interquartile range) according to data distribution. Categorical variables are presented as frequencies and percentages. Comparisons were performed using one-way ANOVA, the Kruskal-Wallis test, or the chi-square test as appropriate. p-values were adjusted for multiple comparisons using the Benjamini-Hochberg false discovery rate; *q* < 0.05 was considered statistically significant.

The prevalence rates of hypertension were higher in the SAP-G, UAP-G, and STEMI-G compared with the control-G. The prevalence rates of diabetes were higher in the UAP-G and STEMI-G than in the control-G. A larger proportion of smoking patients was noted in the UAP-G, NSTEMI-G, and STEMI-G than in the control-G while the smoking rate in the SAP-G was lower than that in the UAP-G and STEMI-G (Table 2).

**Table 2:** Comparison of population characteristics between the control-group and the subsets of the CAD-group.

Index	Control (n = 181)	SAP (n = 57)	UAP (n = 122)	NSTEMI (n = 26)	STEMI (n = 92)
Age (years)	63.74 ± 8.51	64.42 ± 7.43	64.71 ± 9.72	62.65 ± 11.38	61.40 ± 11.27
Female sex (%)	35.12	47.01	40.33	38.14	33.85
BMI (kg/m²)	24.52 ± 3.08	25.28 ± 3.26	24.41 ± 3.21	24.35 ± 2.86	25.34 ± 3.41
Hypertension (%)	42.61	72.15a-b	64.97a-b	61.76	62.18a-b
Diabetes (%)	10.65	24.78	34.65a-b	27.14	31.74a-b
Smoking (%)	22.07	28.29	43.66a-b^,^a-b	42.52a-b	43.66a-b^,^a-b

Notes: CAD, Coronary Artery Disease; SAP, Stable Angina Pectoris; UAP, Unstable Angina Pectoris; NSTEMI, Non-ST-Elevation Myocardial Infarction; STEMI, ST-Elevation Myocardial Infarction; BMI, Body Mass Index. Continuous variables are presented as mean ± standard deviation or median (interquartile range) according to data distribution. Categorical variables are presented as frequencies and percentages. p-values were adjusted for multiple comparisons using the Benjamini-Hochberg false discovery rate; *q* < 0.05 was considered statistically significant.

a-b Indicate a significant difference (p < 0.05) when compared with the control and SAP groups, respectively.

### Comparison of biochemical marker levels between the control-G and CAD-G (overall and subsets)

Elevated High-Sensitivity C-Reactive Protein (HS-CRP) and glycosylated Hemoglobin (HbA1c) levels were higher in the CAD-G than in the control-G. Creatinine levels were higher in the SAP-G, UAP-G, NSTEMI-G, and STEMI-G than in the control-G, but there was no significant difference in creatinine levels among the subsets of the CAD-G (p > 0.05). The NSTEMI and STEMI groups showed higher HS-CRP levels than the control-G, SAP-G, and UAP-G, and increased HbA1c levels were found in the UAP-G and STEMI-G than in the control-G. Moreover, the NSTEMI-G showed elevated HbA1c levels compared to the UAP-G (Table 3, Table 4).

**Table 3:** Comparison of biochemical marker levels between the control and CAD-groups.

Index	Control (n = 181)	CAD (n = 297)	p-value
LDL-C (mmol/L)	2.81 ± 0.90	2.93 ± 0.93	0.42
HDL-C (mmol/L)	1.37 ± 0.39	1.23 ± 0.51	0.06
TG (mmol/L)	1.32 (0.96–1.98)	1.48 (1.08–2.10)	0.60
TC (mmol/L)	4.30 ± 1.18	4.34 ± 1.01	0.95
HS-CRP (mg/L)	1.10 (0.60–2.80)	3.20 (1.20–9.80)ᵃ	<0.01
HbA1c (%)	5.41 ± 0.87	6.15 ± 1.37ᵃ	<0.01
Cr (µmol/L)	63.97 ± 11.86	78.14 ± 35.76	0.062

Notes: LDL-C, Low-Density Lipoprotein Cholesterol; HDL-C, High-Density Lipoprotein Cholesterol; TG, Triglyceride; TC, Total Cholesterol; hs-CRP, High-sensitivity C-Reactive Protein; HbA1c, Glycosylated Hemoglobin; Cr, Creatinine. Normally distributed variables are presented as mean ± standard deviation and compared using the independent-samples *t*-test. Skewed variables, including hs-CRP and TG, are presented as median (interquartile range) and compared using the Mann-Whitney *U test*. Categorical variables are presented as frequencies and percentages and compared using the Chi-Square test or Fisher’s exact test when appropriate. p-values were adjusted for multiple comparisons using the Benjamini-Hochberg false discovery rate; *q* < 0.05 was considered statistically significant.

**Table 4:** Comparison of biochemical marker levels of patients in the control-G and the subsets of the CAD-group.

Index	Control (n = 181)	SAP (n = 57)	UAP (n = 122)	NSTEMI (n = 26)	STEMI (n = 92)
LDL-C (mmol/L)	2.81 ± 0.90	2.81 ± 0.91	2.76 ± 0.94	2.91 ± 0.67	3.16 ± 0.86a-c^,^a-c
HDL-C (mmol/L)	1.37 ± 0.39	1.28 ± 0.31	1.25 ± 0.62	1.37 ± 0.76	1.14 ± 0.26a-c
TG (mmol/L)	1.32 (0.96–1.98)	1.40 (1.02–2.10)	1.38 (0.98–2.05)	1.45 (1.05–2.20)	1.42 (1.00–2.15)
TC (mmol/L)	4.30 ± 1.18	4.32 ± 1.01	4.27 ± 1.10	4.32 ± 0.90	4.45 ± 0.88
HS-CRP (mg/L)	1.10 (0.60–2.80)	2.30 (1.10–5.60)	2.10 (0.90–5.20)	8.50 (4.20–18.60)a-c^,^a-c^,^a-c	6.80 (3.10–15.40)a-c^,^a-c^,^a-c
HbA1c (%)	5.41 ± 0.87	5.93 ± 1.28	6.18 ± 1.34a-c	6.20 ± 1.76a-c	6.25 ± 1.35a-c
Cr (µmol/L)	63.97 ± 11.86	69.85 ± 15.72a-c	70.86 ± 19.01a-c	76.64 ± 34.82a-c	93.21 ± 40.17a-c

Notes: LDL-C, Low-Density Lipoprotein Cholesterol; HDL-C, High-Density Lipoprotein Cholesterol; TG, Triglyceride; TC, Total Cholesterol; hs-CRP, high-sensitivity C-Reactive Protein; HbA1c, Glycosylated Hemoglobin; Cr, Creatinine; SAP, Stable Angina Pectoris; UAP, Unstable Angina Pectoris; NSTEMI, Non-ST-Elevation Myocardial Infarction; STEMI, ST-Elevation Myocardial Infarction.

a-c Indicate a significant difference (p < 0.05) when compared with the control, SAP, and UAP groups, respectively. Normally distributed variables are presented as mean ± standard deviation and compared using one-way ANOVA followed by Tukey’s post hoc test. Skewed variables, including hs-CRP and TG, are presented as median (interquartile range) and compared using the Kruskal-Wallis test followed by Dunn’s post hoc test. Categorical variables are presented as frequencies and percentages and compared using the Chi-Square test or Fisher’s exact test when appropriate. p-values were adjusted for multiple comparisons using the Benjamini-Hochberg false discovery rate; *q* < 0.05 was considered statistically significant.

### Comparison of CD4⁺ and CD8⁺ T-lymphocyte levels and CD4⁺/CD8⁺ ratio between all studied groups

The CD4⁺ counts and CD4⁺/CD8⁺ ratios in the STEMI-G were significantly higher than those in the control-G, SAP-G, and UAP-G, while an increasing non-significant tendency was observed in comparisons with the NSTEMI-G. Moreover, the CD4⁺ counts and CD4⁺/CD8⁺ ratios in the UAP-G were higher than those in the control-G and SAP-G. The CD8⁺ counts in the CAD-G were lower than those in the control-G. Specifically, the CD8⁺ counts in the NSTEMI-G and STEMI-Gs were lower than those in the SAP-G, and were similarly lower in the UAP-G, NSTEMI-G, and STEMI-G than in the control-G (Table 5).

**Table 5:** Comparison of T-lymphocyte counts and CD4⁺/CD8⁺ ratios between the control-group and the subsets of the CAD-group.

Index	Control (n = 181)	SAP (n = 57)	UAP (n = 122)	NSTEMI (n = 26)	STEMI (n = 92)
CD4⁺ (cells/μL)	754.26 ± 218.33	778.61 ± 226.47	819.43 ± 268.15a-c^,^a-c	1004.72 ± 323.61a-c^,^a-c^,^a-c	1025.37 ± 366.84a-c^,^a-c^,^a-c
CD8⁺ (cells/μL)	620.17 ± 209.42	598.45 ± 185.16	549.72 ± 189.63a-c	498.63 ± 187.51a-c^,^a-c	488.42 ± 157.76a-c^,^a-c
CD4⁺/CD8⁺	1.28 ± 0.38	1.38 ± 0.45a-c	1.53 ± 0.36a-c^,^a-c	1.91 ± 0.61a-c^,^a-c^,^a-c	1.98 ± 0.43a-c^,^a-c^,^a-c

Notes: CD4⁺: CD4-positive T-lymphocytes; CD8⁺: CD8-positive T lymphocytes; CAD: coronary artery disease; SAP: stable angina pectoris; UAP: unstable angina pectoris; NSTEMI: non-ST-elevation myocardial infarction; STEMI: ST-elevation myocardial infarction. Continuous variables are presented as mean ± standard deviation or median (interquartile range) according to data distribution. p-values were adjusted for multiple comparisons using the Benjamini-Hochberg false discovery rate; *q* < 0.05 was considered statistically significant.

a-c Indicate a significant difference (p < 0.05) when compared with the control, SAP, and UAP groups, respectively.

### Comparison of SYNTAX scores among patients in each subset of the CAD-G

The SYNTAX scores were higher in the STEMI-G than in the control-G and SAP-G. When compared with the UAP-G and NSTEMI-G, SYNTAX scores in the STEMI-G tended to be higher but the difference was not statistically significant. The SYNTAX scores were significantly higher in both NSTEMI-G and UAP-G than in the control-G and SAP-G (Table 6).

**Table 6:** SYNTAX scores of patients in each group.

Index	Control (n = 181)	SAP (n = 57)	UAP (n = 122)	NSTEMI (n = 26)	STEMI (n = 92)
SYNTAX score	0.32 ± 1.22	4.91 ± 3.18a-b	15.62 ± 13.45a-b^,^a-b	16.83 ± 13.86a-b^,^a-b	20.27 ± 13.14a-b^,^a-b

Notes: SAP, Stable Angina Pectoris; UAP, Unstable Angina Pectoris; NSTEMI, Non-ST-Elevation Myocardial Infarction; STEMI, ST-Elevation Myocardial Infarction. Continuous variables are presented as mean ± standard deviation or median (interquartile range) according to data distribution. p-values were adjusted for multiple comparisons using the Benjamini-Hochberg false discovery rate; *q* < 0.05 was considered statistically significant.

a-b Indicate a significant difference (p < 0.05) when compared with the control and SAP groups, respectively.

### Correlation analysis between SYNTAX score and T-cell subsets

In unadjusted analyses, SYNTAX score was positively correlated with CD4⁺ count (*r* = 0.384, 95% CI 0.282–0.478, p < 0.001), negatively correlated with CD8⁺ count (*r* = −0.340, 95% CI −0.436 – −0.240, p < 0.001), and positively correlated with the CD4⁺/CD8⁺ ratio (*r* = 0.303, 95% CI 0.196–0.403, p < 0.001). The correlation magnitudes were weak to moderate. These correlations remained significant after Benjamini-Hochberg False Discovery Rate (FDR) correction (*q* < 0.001 for all).

After adjustment for age, sex, BMI, hypertension, diabetes, smoking, HbA1c, hs-CRP, and creatinine, the CD4⁺/CD8⁺ ratio remained significantly associated with SYNTAX score (β = 4.88, 95% CI 1.42–8.30, p = 0.006). In the model additionally adjusted for medication use, the association remained significant (β = 4.12, 95% CI 0.78–7.46, p = 0.016).

### Discriminatory performance of the CD4⁺/CD8⁺ ratio for coronary stenosis severity

ROC analysis was restricted to patients with CAD. Patients were categorized into a mild stenosis group with SYNTAX score ≤ 23 (n = 207) and a moderate-to-severe stenosis group with SYNTAX score > 23 (n = 90). The ROC curves for the CD4^+^/CD8^+^ ratio alone, the base clinical model, and the base model plus the ratio are shown in Fig. 1. The AUC for the CD4^+^/CD8^+^ ratio alone in identifying SYNTAX score >23 was 0.598 (95% CI 0.526–0.670). At the Youden-optimal cutoff of 1.90, sensitivity was 0.587, specificity was 0.552, positive predictive value was 0.36, and negative predictive value was 0.76. The Youden index was 0.139, indicating limited discrimination when used alone.

**Fig. 1:**
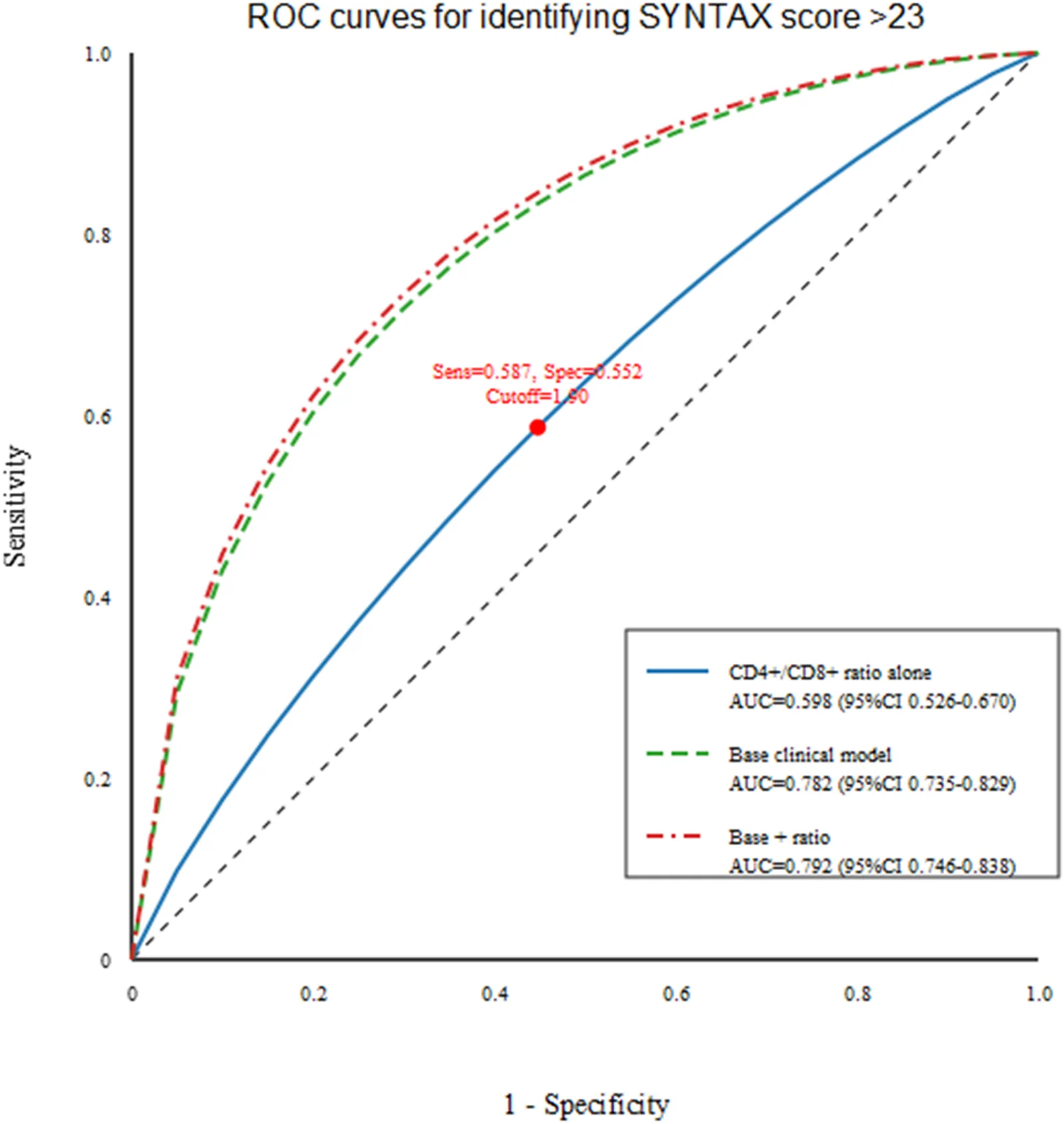
Receiver Operating Characteristic (ROC) curves for identifying SYNTAX score > 23 in patients with CAD. Blue line, CD4⁺/CD8⁺ ratio alone; green dashed line, base clinical model; red dash-dot line, base clinical model plus CD4⁺/CD8⁺ ratio. The red dot indicates the Youden-optimal cutoff for the ratio. AUC, Area Under the Curve; CI, Confidence Interval.

When the CD4⁺/CD8⁺ ratio was added to a base model containing conventional risk factors, the AUC changed from 0.782 (95% CI 0.735–0.829) to 0.792 (95% CI 0.746–0.838), with ΔAUC = 0.010, DeLong p = 0.43, NRI = 0.082, and IDI = 0.006. These findings suggest that the ratio should not be used as a standalone diagnostic test.

For SYNTAX score > 23, the unadjusted OR per 1-unit increase in the CD4⁺/CD8⁺ ratio was 2.85 (95% CI 1.45–5.60, p = 0.002). Per 0.1-unit increase, the OR was 1.11 (95% CI 1.04–1.19, p = 0.002). After full adjustment, the OR was 2.31 (95% CI 1.12–4.76, p = 0.023).

## Discussion

The initiation and progression of Atherosclerosis (AS) are associated with local accumulation of toxic lipids and a series of complex immune responses.^2^ Recent studies have shown many immune cells, such as macrophages, Dendritic Cells (DCs), T-lymphocytes, and B-lymphocytes, at AS sites, all of which can influence the pathological progression of AS by secreting pro- and anti-inflammatory cytokines and chemokines.^3^ Important information about the activity of the adaptive immune response in the coronary vasculature can be obtained by quantifying CD4⁺ and CD8⁺ T-lymphocyte subsets in patients with CAD. The results of this study showed an elevated CD4⁺/CD8⁺ ratio in patients with CAD. This finding could be a result of the migration of effector T cells from peripheral blood into the reperfused myocardium mediated by abnormal chemokine levels, such as fractalkine. It was initially believed that only macrophages were present in AS plaques. It has now been demonstrated that AS plaques also contain T-lymphocytes and their subsets, monocytes, and DCs, which play important roles in the formation and rupture of AS plaques.^4^^,^^12^ Current clinical tests for T-lymphocyte subset detection allow precise quantification of CD4⁺ and CD8⁺ levels and percentages, both of which are involved in the immune response and its regulation. Experiments in rodent models suggested that activation of T-helper 1 (Th1) cells might play a key role in the progression of AS.^5^ Th1 cell-related cytokines, such as IFN-γ, have been found in advanced AS lesions in mice.^13^ In mice fed a high-fat diet, depletion of CD4⁺ reduced the size of aortic plaques.^14^ Moreover, CD4⁺ cells within human AS plaques were found to secrete a high level of IFN-γ.^15^ These studies suggested a pro-AS role for CD4⁺. It has also been shown that activated CD8⁺ cells could secrete large amounts of pro-inflammatory cytokines, which activate macrophages to release a variety of cytokines that initiate immune cascades and thus promote AS.^7^

In this study, we found that CD4⁺ counts were significantly higher in the NSTEMI and STEMI groups, while CD8⁺ counts were significantly lower, than those in the other groups. In patients with CAD, CD4⁺ levels were significantly higher in the STEMI group than in the NSTEMI-G, whereas the NSTEMI-G showed slightly higher CD4⁺ levels compared with the SAP-G. The CD4⁺ levels were slightly higher in the SAP group than in the control-G. The CD4⁺/CD8⁺ ratios were significantly higher in the SAP, UAP, NSTEMI, and STEMI groups compared with the control-G, which is consistent with findings from previous studies.^6^ These results confirmed that the in vivo immune/inflammatory response was enhanced during the progression of coronary artery lesions, suggesting that patients with CAD are in a state of immune dysfunction with abnormal T-lymphocyte subset ratios.

Most recent studies have focused on investigating the relationship between AS and T lymphocyte subsets.^8^^,^^10^ The SYNTAX score is used to assess the incidence rate of cardiovascular events in the following year in patients undergoing percutaneous coronary intervention and can be an effective tool for predicting the occurrence of major ischemic events in patients with stable ischemic heart disease or multivessel/left main CAD.^16^^,^^17^ In this study, we found an association between T-lymphocyte subset counts in peripheral blood and the SYNTAX score, which could be useful in assessing the severity of coronary stenosis in patients with CAD. Specifically, the SYNTAX score was positively correlated with both CD4⁺ counts and the CD4⁺/CD8⁺ ratio, and negatively correlated with CD8⁺ counts. However, the correlation coefficients were only weak to moderate, and the cross-sectional design precludes causal inference. Therefore, this study demonstrates the potential significance of measuring T-lymphocyte subset levels in patients with CAD and indicates that the CD4⁺/CD8⁺ ratio may serve as a complementary candidate marker for assessing the severity of coronary stenosis. Although the CD4⁺/CD8⁺ ratio showed a significant association with high SYNTAX scores, its modest sensitivity and specificity suggest that it is not yet robust enough to serve as a standalone diagnostic tool in clinical practice. It may, however, provide additive value when used in conjunction with traditional risk factors and scoring systems, pending confirmation in larger studies.

Previous studies have shown that metabolic and biochemical markers are associated with angiographic CAD severity. For example, the visceral adiposity index has been associated with increased SYNTAX score in patients with type 2 diabetes mellitus, and the gamma-glutamyl transferase to albumin ratio has been reported to predict coronary artery disease severity assessed by coronary computed tomography angiography.^18^^,^^19^ These findings support the concept that coronary anatomical burden is multifactorial and cannot be fully captured by a single biomarker. Our study extends this line of investigation by focusing on the adaptive immune axis, specifically the peripheral CD4⁺/CD8⁺ T-cell balance.

The SYNTAX score provides a reproducible anatomical assessment of coronary lesion complexity; however, it does not incorporate clinical variables. The SYNTAX score II was developed to integrate anatomical complexity with clinical characteristics and has been shown to improve risk assessment in selected high-risk populations, including patients with cardiogenic shock undergoing primary percutaneous coronary intervention.^20^ This reinforces the need to interpret immune biomarkers such as the CD4⁺/CD8⁺ ratio together with anatomical and clinical variables rather than in isolation. Future research may also explore artificial intelligence and machine-learning approaches that integrate inflammatory markers, immune-cell profiles, electrocardiographic features, clinical comorbidities, and angiographic data for individualized prediction and management of obstructive CAD.^21^^,^^22^

Several limitations of this study should be acknowledged. First, the cross-sectional design precludes any causal inference regarding the relationship between T-cell subset imbalance and CAD severity. Second, this was a single-center study with a relatively modest sample size, which may limit the generalizability of our findings. Third, the control group consisted of patients who underwent coronary angiography and had no obstructive lesions, which may introduce selection bias. Fourth, despite multivariable adjustment, residual confounding by unmeasured variables, including detailed medication history, duration of diabetes or hypertension, and lifestyle factors, cannot be excluded. Fifth, the modest sensitivity and specificity of the CD4⁺/CD8⁺ ratio observed in the ROC analysis indicate that it should not be used as a standalone diagnostic test, but rather as a complementary biomarker. Sixth, our assessment of T-cell subsets was limited to peripheral blood and may not fully reflect the local immune microenvironment within coronary plaques. Finally, the ROC result requires reanalysis and external validation; the AUC, cutoff, and incremental value should be interpreted cautiously. Future large-scale, prospective, multicenter studies are warranted to validate our findings and to explore the dynamic changes of T-cell subsets over time and their association with long-term clinical outcomes.

## Conclusions

This study demonstrates that patients with CAD exhibit a shift in peripheral T-cell subsets, characterized by elevated CD4⁺ counts, reduced CD8⁺ counts, and an increased CD4⁺/CD8⁺ ratio, most pronounced in acute coronary syndromes. The CD4⁺/CD8⁺ ratio was positively associated with SYNTAX score, but the correlation was weak to moderate. The ratio showed limited discriminatory ability for moderate-to-severe stenosis and did not clearly provide substantial incremental value beyond conventional risk factors. Therefore, the CD4⁺/CD8⁺ ratio should be considered a complementary candidate marker associated with angiographic complexity, not a standalone diagnostic or predictive tool. The cross-sectional design, single-center sample, residual confounding, and selection bias of the angiography-based control group preclude causal inference and limit generalizability. Larger prospective multicenter studies with multivariable adjustment and external validation are warranted.

## Abbreviations

CAD, Coronary Artery Disease; CA, Coronary Artery; SAP, Stable Angina Pectoris; UAP, Unstable Angina Pectoris; NSTEMI, Non-ST-Elevation Myocardial Infarction; STEMI, ST-Elevation Myocardial Infarction; %DS, Percentage Diameter Stenosis; hs-CRP, High-sensitivity C-Reactive Protein; HbA1c, Glycosylated Hemoglobin; Cr, Creatinine; LDL-C, Low-Density Lipoprotein Cholesterol; HDL-C, High-Density Lipoprotein Cholesterol; TG, Triglyceride; TC, Total Cholesterol; AS, Atherosclerosis; ROC, Receiver Operating characteristic; AUC, Area Under the Curve; FDR, False Discovery Rate; NRI, Net Reclassification Improvement; IDI, Integrated Discrimination Improvement.

## Ethics approval and consent to participate

The study was performed in accordance with the ethical standards of the 1964 Declaration of Helsinki and its later amendments or comparable ethical standards. Approval was obtained from the Ethics Committee of Affiliated Hospital of Yanbian University (Yanbian Hospital) (approval no 2019,135, date of approval: September 15, 2019). Written informed consent was obtained from all individual participants included in the study.

## Consent for publication

Not applicable. This manuscript does not contain any individual person’s data in any form (including individual details, images, or videos).

## Clinical trial number

Not applicable.

## Authors’ contributions

Wenhao Li: Writing-original draft; writing-review & editing; formal analysis; investigation; data curation; visualization.

Chunzi Jin: Writing-original draft; writing-review & editing; formal analysis; investigation; data curation.

Meina Piao: Investigation; data curation.

Guangming Jin: Supervision; validation; resources.

Xiang Li: Conceptualization; supervision; project administration; funding acquisition.

All authors read and approved the final manuscript.

## Funding

This work was supported by the National Natural Science Foundation of China under grant numbers 82060052 and 82160062. The funders had no role in the study design, data collection, analysis, decision to publish, or preparation of the manuscript.

## Conflicts of interest

The authors declare no conflicts of interest.

## Data Availability

The datasets used and/or analyzed during the current study are available from the corresponding author on reasonable request.
